# The effect of statin therapy on heart failure events: a collaborative meta-analysis of unpublished data from major randomized trials

**DOI:** 10.1093/eurheartj/ehv072

**Published:** 2015-03-23

**Authors:** David Preiss, Ross T. Campbell, Heather M. Murray, Ian Ford, Chris J. Packard, Naveed Sattar, Kazem Rahimi, Helen M. Colhoun, David D. Waters, John C. LaRosa, Pierre Amarenco, Terje R. Pedersen, Matti J. Tikkanen, Michael J. Koren, Neil R. Poulter, Peter S. Sever, Paul M. Ridker, Jean G. MacFadyen, Scott D. Solomon, Barry R. Davis, Lara M. Simpson, Haruo Nakamura, Kyoichi Mizuno, Rosa M. Marfisi, Roberto Marchioli, Gianni Tognoni, Vasilios G. Athyros, Kausik K. Ray, Antonio M. Gotto, Michael B. Clearfield, John R. Downs, John J. McMurray

**Affiliations:** 1 BHF Glasgow Cardiovascular Research Centre, University of Glasgow, 126 University Place, Glasgow G12 8TA, UK; 2 Robertson Centre for Biostatistics, University of Glasgow, Glasgow, UK; 3 Glasgow Clinical Research Facility, Western Infirmary, Glasgow, UK; 4 George Institute for Global Health, University of Oxford, Oxford, UK; 5 Medical Research Institute, University of Dundee, Dundee, UK; 6 Department of Medicine, University of California, San Francisco, CA, USA; 7 SUNY Health Science Center at Brooklyn, New York, NY, USA; 8 Department of Neurology and Stroke Center, Bichat University Hospital, Paris, France; 9 University of Oslo and Centre for Preventative Medicine, Oslo University Hospital, Ullevål, Oslo, Norway; 10 University of Helsinki and Heart and Lung Center, Helsinki University Central Hospital and Folkhälsan Research Center, Helsinki, Finland; 11 Jacksonville Center for Clinical Research, Jacksonville, FL, USA; 12 International Center for Circulatory Health, National Heart & Lung Institute, Imperial College London, London, UK; 13 Division of Cardiovascular Medicine, Department of Medicine, Brigham and Women's Hospital, Harvard Medical School, Boston, MA, USA; 14 The University of Texas School of Public Health, Houston, TX, USA; 15 Mitsukoshi Health and Welfare Foundation, Shinjuku-ku, Tokyo, Japan; 16 Department of Medicine, Nippon Medical School, Bunkyo-ku, Tokyo, Japan; 17 Consorzio Mario Negri Sud, Santa Maria Imbaro, Chieti, Italy; 18 Second Propedeutic Department of Internal Medicine, Medical School, Aristotle University of Thessaloniki, Hippokration Hospital, Thessaloniki, Greece; 19 Weill Cornell Medical College, New York, NY, USA; 20 College of Osteopathic Medicine, Touro University, Vallejo, CA, USA; 21 Department of Medicine, University of Texas Health Science Center, San Antonio, TX, USA; 22 The South Texas Veterans Health Care System, San Antonio, TX, USA

**Keywords:** Statin, Heart failure, Randomized trial, Prevention, Meta-analysis

## Abstract

**Aims:**

The effect of statins on risk of heart failure (HF) hospitalization and HF death remains uncertain. We aimed to establish whether statins reduce major HF events.

**Methods and results:**

We searched Medline, EMBASE, and the Cochrane Central Register of Controlled Trials for randomized controlled endpoint statin trials from 1994 to 2014. Collaborating trialists provided unpublished data from adverse event reports. We included primary- and secondary-prevention statin trials with >1000 participants followed for >1 year. Outcomes consisted of first non-fatal HF hospitalization, HF death and a composite of first non-fatal HF hospitalization or HF death. HF events occurring <30 days after within-trial myocardial infarction (MI) were excluded. We calculated risk ratios (RR) with fixed-effects meta-analyses. In up to 17 trials with 132 538 participants conducted over 4.3 [weighted standard deviation (SD) 1.4] years, statin therapy reduced LDL-cholesterol by 0.97 mmol/L (weighted SD 0.38 mmol/L). Statins reduced the numbers of patients experiencing non-fatal HF hospitalization (1344/66 238 vs. 1498/66 330; RR 0.90, 95% confidence interval, CI 0.84–0.97) and the composite HF outcome (1234/57 734 vs. 1344/57 836; RR 0.92, 95% CI 0.85–0.99) but not HF death (213/57 734 vs. 220/57 836; RR 0.97, 95% CI 0.80–1.17). The effect of statins on first non-fatal HF hospitalization was similar whether this was preceded by MI (RR 0.87, 95% CI 0.68–1.11) or not (RR 0.91, 95% CI 0.84–0.98).

**Conclusion:**

In primary- and secondary-prevention trials, statins modestly reduced the risks of non-fatal HF hospitalization and a composite of non-fatal HF hospitalization and HF death with no demonstrable difference in risk reduction between those who suffered an MI or not.

**See page 1502 for the editorial comment on this article (doi:10.1093/eurheartj/ehv133)**

## Introduction

Heart failure (HF) is a condition characterized by debilitating symptoms, a particularly poor quality of life, frequent hospital admissions and reduced survival.^1^ It also places a major economic burden on healthcare systems. Consequently, therapies which reduce the risk of developing HF are highly desirable and likely to be cost effective. Coronary heart disease is reported to be the most common cause of HF.^2^ Statin therapy has been conclusively shown to reduce the risk of myocardial infarction (MI) in primary- and secondary-prevention populations.^3^ By reducing first and recurrent injurious myocardial events, statin therapy should reduce the development of HF. It is also possible that statin therapy may influence the development of HF by other mechanisms.

To date, about half of the major placebo- and standard care-controlled statin endpoint trials have published data on a variety of HF outcomes and a borderline reduction in HF hospitalization or HF death was only shown in one trial.^4–11^ Data regarding HF hospitalization from four dose-comparison trials have been pooled with the overall result suggesting that intensive statin therapy reduces HF hospitalization compared with moderate dose therapy.^12^ However, the types of HF endpoints have varied somewhat with some trials reporting any HF adverse event, some reporting HF death, some reporting non-fatal HF hospitalization, and some reporting a composite of fatal and non-fatal HF events. This heterogeneity in the categories of HF outcomes collected, along with missing information, has precluded a comprehensive pooling of HF data. In addition, none of the existing reports described whether any effect on HF hospitalization might be fully explained by preventing precursor MIs.

The purpose of this analysis was to obtain comprehensive and harmonized data for major HF events (non-fatal hospitalization, death, and a composite of both) in major statin trials for the purpose of evaluating whether, and if so to what extent, statin therapy may reduce major HF events. In addition, we wished to explore whether any reduction in HF was primarily driven by a reduction in non-fatal MI.

## Methods

### Data sources and searches

We collected data from major randomized controlled trials designed to assess the effect of statins on cardiovascular outcomes. We searched Medline, Embase, and the Cochrane Central Register of Controlled Trials with the terms ‘statin’ and ‘HMG CoA reductase inhibitor’, plus individual statin names as title or keywords, on 25th March 2014.

### Study selection

We limited the search to trials of adults, published in the English language, between 1 January 1994 and 1 January 2014. We included placebo controlled, standard care-controlled, and intensive  vs. moderate dose trials in primary prevention, secondary-prevention, and mixed primary- and secondary-prevention populations. We excluded trials conducted entirely in participants with HF, dialysis, or organ transplant populations, those with <1000 enrolled participants, trials with a mean follow-up of <1 year, trials of combination therapy, and trials assessing surrogate markers of cardiovascular disease. Study protocol and data request sheets were sent to investigators from the relevant trials as described below to collect unpublished data (Supplementary material online, *Appendix*). A specific definition of HF events was not provided to the contributing investigators.

### Data extraction and quality assessment

**Figure 1:**
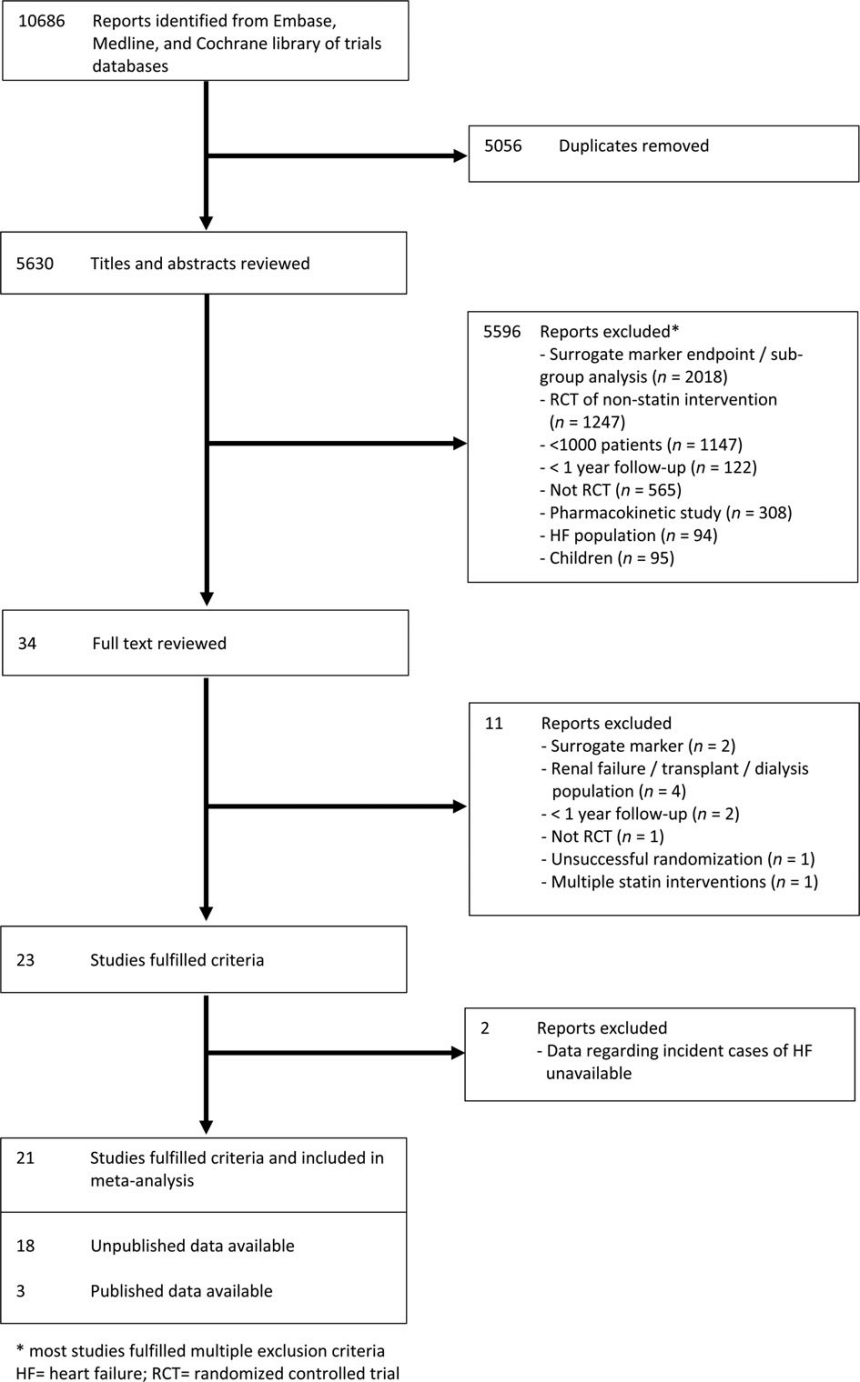
Flow diagram of literature search to identify randomized placebo-controlled trials, standard care-controlled trials, and dose-comparison statin trials.

Two researchers (R.C. and D.P.) independently reviewed abstracts and manuscripts to identify relevant trials. A third reader (J.J.M.) resolved any discrepancies. An assessment of study design and quality for each trial was made using the Jadad score.^13^ Our search strategy identified 23 potentially suitable trials for which unpublished data were sought^6–11,14–30^ (*Figure 1*). The study was conducted according to published Preferred Reporting Items for Systematic Reviews and Meta-Analyses guidelines.^31^ Unpublished tabular data were collected regarding the numbers of participants who experienced a non-fatal hospitalization for HF, the number that died due to HF, and the number that suffered either a non-fatal HF hospitalization or HF death (composite HF outcome) in each arm. Investigators were asked to detail the number of participants with HF at randomization in both treatment arms (where available), LDL-cholesterol levels at randomization and 1 year, and the number of participants who suffered a within-trial non-fatal MI. Numbers with incident HF events were also requested separately in only those participants without HF at baseline. Heart failure events occurring <30 days after within-trial MI were specifically excluded from unpublished data sought from collaborating trials. This decision was driven by the following considerations: events within 30 days of MI would have been variably recorded as many participants would still have been in hospital due to the index MI (i.e. some events would still not be captured by assessing HF hospitalization); development of HF soon after MI may be transient, reflecting cardiac stunning, and may not necessarily lead to chronic HF; and if participants developed clinically significant HF they may well have suffered a subsequent HF hospitalization in the trial anyway. Of the 23 trials, 17 trials^7–11,15,16,18,20,21,24–30^ were able to provide unpublished HF data, with HF events occurring <30 days after within-trial MI excluded. These results were analysed for the main HF analyses. For the remaining six trials, HF outcomes were available from four trials (three with published data;^6,14,23^ one with unpublished data^17^). Data regarding timing of incident cases of HF with regard to within-trial (i.e. post-randomization) MI were not available in these four trials with the result that HF events within 30 days of MI could not be excluded and their results were therefore only combined with the full dataset in sensitivity analyses. Two authors (D.P. and R.C.) independently abstracted and tabulated information about the number of participants without HF at baseline and incident cases of HF according to randomization group in those trials where only published data were available.

### Data synthesis and analysis

To identify potential relationships of statins with the risk of developing HF, we calculated risk ratios (RRs) as the ratio of cumulative incidence and 95% confidence intervals (CIs) from the available data for all trial participants at baseline and for those who experienced an HF event during trial follow-up. Study-specific RRs were pooled using fixed-effects model meta-analysis. Statistical heterogeneity across studies was quantified using the *I*^2^ statistic, with *P* > 0.10 considered statistically non-significant. The *I*^2^ statistic is derived from the *Q* statistic ([*Q*− d*f*/*Q*] × 100) and provides a measure of the proportion of the overall variation attributable to between-study heterogeneity. We also separately analysed the risk of HF events depending on whether a participant had or had not suffered a preceding within-trial MI. However, the available data did not allow a formal statistical comparison of those who had and had not suffered a preceding within-trial MI.

Placebo- and standard care-controlled statin trials as well as dose-comparison statin trials were combined in all analyses though their respective pooled results were also compared by fixed-effect inverse-variance modelling in a sensitivity analysis. Additional sensitivity analyses included random effects meta-analysis of non-fatal HF hospitalization, a comparison of trial populations (primary prevention, secondary prevention, and mixed population), a comparison of trials which had and had not pre-specified any HF outcome, an analysis limited to only those trials which could provide data on participants without HF at baseline, and an analysis which added HF events from four trials (including events within 30 days of within-trial MI^6,14,17,23^) to the unpublished trial data already analysed in the main analyses. We assessed the potential for publication bias through formal statistical testing, namely funnel plots and Egger tests.^32^ To evaluate potential relationships of the effects of statins on non-fatal MI and LDL-cholesterol reductions, respectively, with occurrence of HF events, meta-regression analyses were performed.

All *P*-values were two sided, and *P*-value of <0.05 was considered statistically significant for the meta-analyses and meta-regression analyses. Analyses were conducted using Stata/SE 13 (StataCorp).

## Results

In the main analyses, we included unpublished data from up to 132 568 participants (average age 63 years, 29% women) from 17 trials who were followed up for 4.3 (weighted SD 1.4) years on average. These participants included a small proportion with symptomatically mild HF at baseline (weighted mean 2.8% of participants, range 0–16.1% in 13 trials with available data). Baseline characteristics are provided in *Table 1*. At 1 year, LDL-cholesterol was reduced by a weighted mean of 0.97 mmol/L (weighted SD 0.38 mmol/L). Trials were generally of high quality with a median Jadad score of 5 (range 3–5).

**Table 1:** Baseline characteristics for 21 statin trials providing data on heart failure events

Trial	Year	Trial population	Active arm (*n*)	Control arm (*n*)	Age (years)	LDL-c (mmol/L)	Follow-up (years)	Women (%)	Percentage with HF at baseline (%)	Was HF a pre-specified endpoint?	Included in main HF analyses
4S^5,14^	1994	Secondary	Simvastatin 10–40 mg (2221)	Placebo (2223)	58.5 (−)	4.87 (0.66)	5.4 (−)*	19	–	No	No
WOSCOPS^15^	1995	Primary	Pravastatin 40 mg (3302)	Placebo (3293)	55.2 (5.5)	4.96 (0.45)	4.81 (0.78)	0	0.0	No	Yes
CARE^16^	1996	Secondary	Pravastatin 40 mg (2081)	Placebo (2078)	58.6 (9.3)	3.59 (0.38)	4.83 (0.94)	14	7.2	Yes	Yes
AFCAPS TEXCAPS^17^	1998	Primary	Lovastatin 20–40 mg (3304)	Placebo (3301)	58 (7)	3.89 (0.43)	5.2 (0.9)	15	0.0	Yes	No
LIPID^6^	1998	Secondary	Pravastatin 40 mg (4512)	Placebo (4502)	62 (−)*	3.88 (3.36–4.40)*	6.1 (−)	17	–	Yes	No
GISSI PREVENZIONE^18^	2000	Secondary	Pravastatin 20 mg (2138)	No treatment (2133)	59.9 (10.4)	3.92 (0.67)	1.9 (0.6)	14	16.1	No	Yes
HPS^20^	2002	Mixed	Simvastatin 40 mg (10 269)	Placebo (10 267)	64 (8.4)	3.38 (0.83)	5 (1.1)	25	–	No	Yes
PROSPER^7^	2002	Mixed	Pravastatin 40 mg (2891)	Placebo (2913)	75.3 (3.4)	3.79 (0.80)	3.2 (0.63)	52	–	Yes	Yes
GREACE^8^	2002	Secondary	Atorvastatin 10–80 mg (800)	Usual care (800)	59 (12)	4.65 (0.62)	3 (1)	22	–	Yes	Yes
ALLHAT-LLT^9^	2002	Mixed	Pravastatin 40 mg (5170)	Usual care (5185)	66.6 (7.6)	3.76 (0.55)	4.8 (1.3)	49	0.0	Yes	Yes
ASCOT-LLA^10^	2003	Primary	Atorvastatin 10 mg (5134)	Placebo (5106)	62.7 (8.5)	3.44 (0.72)	3.2 (0.6)	19	0.0	Yes	Yes
CARDS^21^	2004	Primary	Atorvastatin 10 mg (1428)	Placebo (1410)	61.6 (8.1)	3.03 (0.71)	3.84 (1.06)	32	0.1	No	Yes
ALLIANCE^11^	2004	Secondary	Atorvastatin 10–80 mg (1217)	Usual care (1225)	61.2 (8.8)	3.80 (0.68)	4.29 (1.5)	18	6.8	Yes	Yes
A TO Z^23^	2004	Secondary	Simvastatin 40–80 mg (2265)	Placebo + Simvastatin 20 mg (2232)	61 (53–69)*	2.87 (2.46–3.39)*	2.0 (−)*	24	–	Yes	No
TNT^24^	2005	Secondary	Atorvastatin 80 mg (4995)	Atorvastatin 10 mg (5006)	61 (8.8)	2.52 (0.45)	4.9 (0.76)	19	7.8	Yes	Yes
IDEAL^25^	2005	Secondary	Atorvastatin 80 mg (4439)	Simvastatin 20–40 mg (4449)	61.7 (9.5)	3.14 (0.90)	4.62 (0.82)	19	6.0	Yes	Yes
ASPEN^26^	2006	Mixed	Atorvastatin 10 mg (1211)	Placebo (1199)	61 (8.2)	2.93 (0.66)	4 (0.55)	34	0.4	Yes	Yes
MEGA^27^	2006	Primary	Pravastatin 10–20 mg (3866)	Usual care (3966)	58.3 (7.2)	4.05 (0.45)	5.29 (1.92)	68	0.1	No	Yes
SPARCL^28^	2006	Primary	Atorvastatin 80 mg (2365)	Placebo (2366)	62.8 (11.3)	3.45 (0.62)	4.8 (1.1)	40	0.4	Yes	Yes
JUPITER^29^	2008	Primary	Rosuvastatin 20 mg (8901)	Placebo (8901)	66.1 (7.7)	2.70 (0.48)	2.1 (0.9)	38	0.3	No	Yes
SEARCH^30^	2010	Secondary	Simvastatin 80 mg (6031)	Simvastatin 20 mg (6033)	64.2 (8.9)	2.50 (0.61)	6.7 (1.5)	17	–	No	Yes

HF, heart failure; LDL-c, low-density lipoprotein cholesterol.

Data are listed as mean (SD) or *median (interquartile range).

### Effect of statin therapy on non-fatal myocardial infarction

In 16 trials (one trial not able to provide data on non-fatal MI), risk of non-fatal myocardial MI was reduced by 26% on statin therapy (2287 first events in 65 438 participants on statin vs. 3107 first events in 65 530 on control; RR 0.74, 95% CI 0.70–0.78).

### Effect of statin therapy on heart failure outcomes

**Figure 2:**
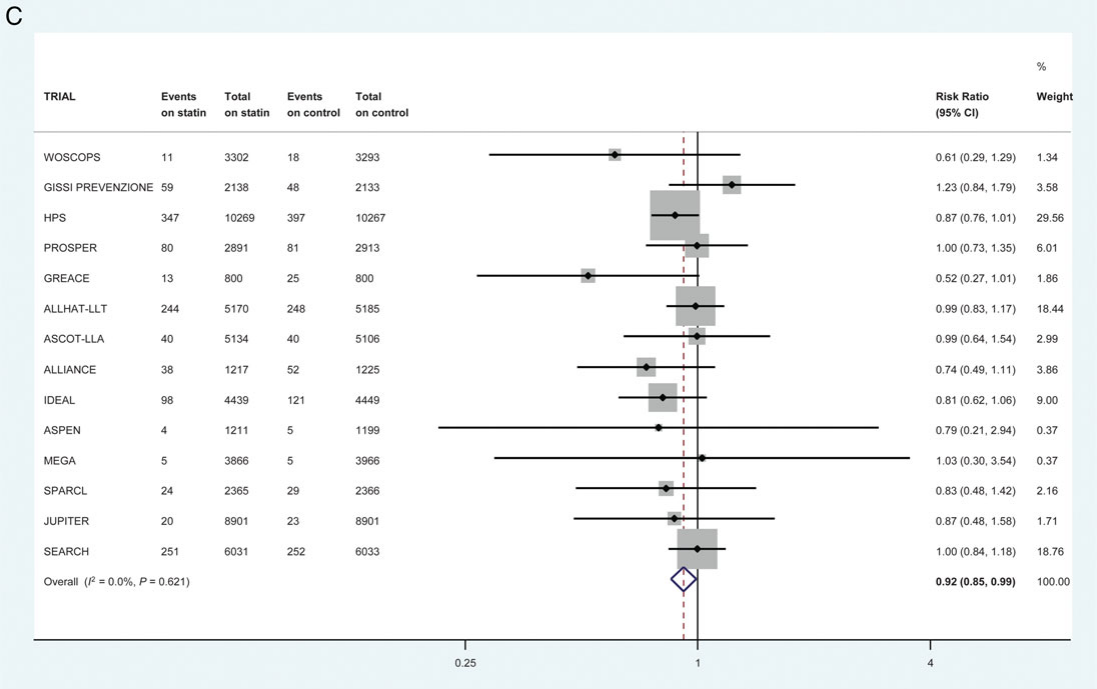
The effect of statin therapy on the risk of (*A*) first non-fatal heart failure hospitalization in 17 trials (*B*) heart failure death in 14 trials (*C*) first composite heart failure outcome in 14 trials. All heart failure events within 30 days of myocardial infarction were excluded.

**Figure 3:**
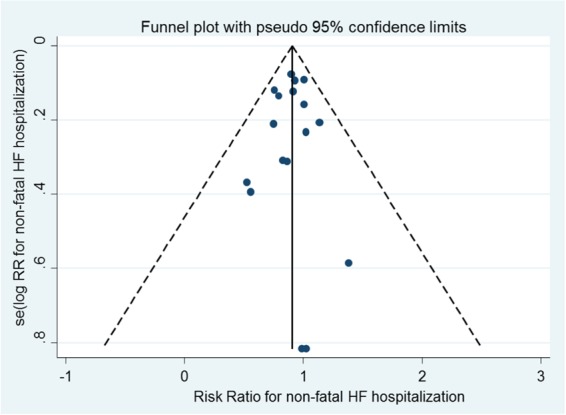
Funnel plot for non-fatal heart failure hospitalization. Egger test *P* = 0.74.

First non-fatal HF hospitalization was reduced by 10% on statins (1344/66 238 vs. 1498/66 330 first events; RR 0.90, 95% CI 0.84–0.97) in 17 trials (*Figure 2A*). This equates to numbers needed to treat of 1454 (non-significant) in the primary prevention trials, 552 (non-significant) in the mixed trials and 200 (95% CI 126–574) in the secondary-prevention trials over 5 years to prevent one patient experiencing a first non-fatal HF hospitalization when the overall control-arm risks of events were ∼1.2, 7.1, and 7.0 per 1000 patient-years, respectively. Heart failure death was not reduced by statin therapy in 14 trials (213/57 734 vs. 220/57 836 events; RR 0.97, 95% CI 0.80–1.17) (*Figure 2B*). The risk of a participant suffering a first composite HF outcome (death or non-fatal hospitalization) was reduced by 8% on statin therapy in 14 trials (1234/57 734 vs. 1344/57 836 first events; RR 0.92, 95% CI 0.85–0.99) (*Figure 2C*). There was little statistical evidence of heterogeneity for all these analyses (*I*^2^ 0%) and there was no statistical evidence of publication bias (*Figure 3*).

**Figure:**
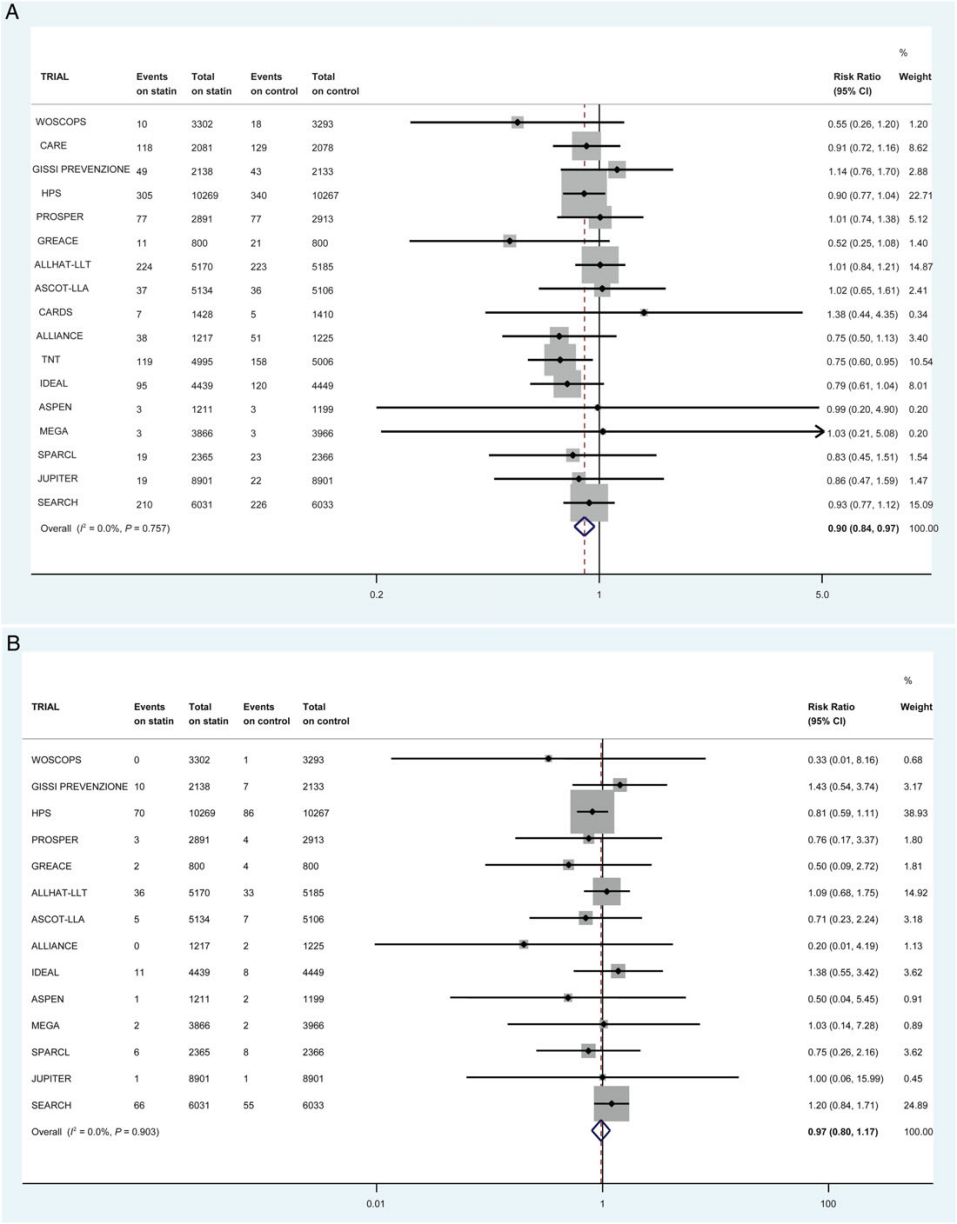

In meta-regression analyses, no relationship was observed between either non-fatal MI reduction (*P* = 0.69; Supplementary material online, *Figure S1*) or absolute LDL-cholesterol reduction achieved at 1 year (*P* = 0.75; Supplementary material online, *Figure S2*) on statins, and risk of first non-fatal HF hospitalisation. Results were similar when the relationships between these two variables were compared with risk of the composite HF outcome (*P* = 0.18 and *P* = 0.18, respectively).

Of the 17 trials included in the main HF analyses, 13 trials were able to provide data on HF status at baseline. Analyses in the 90 001 (97.2% of total) participants without documented HF at baseline in these 13 trials generally produced similar results to the main analyses described above (Supplementary material online, *Table S1*) although with fewer events available for pooling and consequent loss of power, the benefit of statin therapy over control therapy did not reach statistical significance.

### Influence of statin therapy on risk of heart failure events preceded or not preceded by myocardial infarction

Only 10–15% of first composite HF outcomes as well as non-fatal HF hospitalizations were preceded by a documented within-trial non-fatal MI (this excludes all HF events within 30 days of MI). There was no demonstrable difference between the effect of statin therapy on risk of a first HF event preceded by a within-trial MI or not (*Table 2*). For non-fatal hospitalization, RRs were 0.87 (95% CI 0.68–1.11) and 0.91 (95% CI 0.84–0.98) while for the composite HF outcome RRs were 0.86 (95% CI 0.68–1.09) and 0.94 (95% CI 0.86–1.01), respectively.

**Table 2:** Effect of statin therapy on heart failure events according to the presence or absence of a previous within-trial myocardial infarction

	Number of trials	First events/*n* on statin, first events/*n* on control therapy	Risk ratio (95% CI)	*I*^2^ (%)
**Non-fatal heart failure hospitalization^a^**				
First non-fatal hospitalization	17	1344/66 238, 1498/66 300	0.90 (0.84–0.97)	0
No MI preceding first non-fatal hospitalization	15	1096/63 357, 1211/63 452	0.91 (0.84–0.98)	0
MI preceding first non-fatal hospitalization	15	119/63 357, 137/63 452	0.87 (0.68–1.11)	0
**Heart failure death^a^**				
Death	14	213/57 734, 220/57 836	0.97 (0.80–1.17)	0
No MI preceding death	13	178/56 934, 181/57 036	0.98 (0.80–1.21)	0
MI preceding death	7	33/33 124, 35/33 232	0.95 (0.59–1.51)	0
**Heart failure composite outcome (death or non-fatal hospitalization)^a^**				
First composite outcome	14	1234/57 734, 1344/57 836	0.92 (0.85–0.99)	0
No MI preceding first composite outcome	13	1093/56 934, 1170/57 036	0.94 (0.86–1.01)	0
MI preceding first composite outcome	12	128/48 033, 149/48 135	0.86 (0.68–1.09)	0

^a^All HF events occurring <30 days after within-trial MI excluded from analysis.

### Sensitivity analyses

There was no evidence of any difference in the effect of statin therapy on HF events in primary prevention, secondary prevention, and mixed trial populations (*P* = 0.42; Supplementary material online, *Figure S3*). Similarly, no difference was demonstrated between placebo/standard care-controlled trials and dose-comparison trials (*P* = 0.23; Supplementary material online, *Figure S4*). We tested whether there was any heterogeneity between trials which did or did not pre-specify any HF event as a secondary or tertiary outcome and, again, no difference was found (*P* = 0.62; Supplementary material online, *Figure S5*). When random effects meta-analysis was employed for non-fatal HF hospitalization, results were identical to those obtained from fixed-effect meta-analysis, namely RR 0.90 (95% CI 0.84–0.97).

The analyses were repeated with the inclusion of data from the four trials (additional 24 560 participants) which did not report timing of within-trial MI. Results were similar to the main analyses (Supplementary material online, *Table S2*).

## Discussion

This study demonstrates that statin therapy led to reductions in the number of patients suffering HF events in major primary- and secondary-prevention trials over ∼4 years of follow-up. The risks of both non-fatal hospitalization and the composite HF outcome (non-fatal HF hospitalization or HF death) were reduced by ∼10%, driven by a reduction in non-fatal hospitalization. This modest benefit appeared similar in those with and without a history of coronary heart disease at baseline, and across both placebo- and standard care-controlled trials, as well as dose-comparison trials. In contrast, no reduction in HF death was noted though there were fewer events and a small treatment benefit cannot be excluded. While relative risk reductions for HF hospitalizations on statins were similar in primary- and secondary-prevention trial participants, the absolute risk reduction was considerably greater in secondary-prevention participants whose HF event rates were substantially higher.

Pooled results for HF hospitalizations were previously available from four dose-comparison statin trials (*n* = 27 546) in participants with established coronary heart disease, some of whom had HF at baseline. These results published in 2006, which included HF events within 30 days of MI, showed a 27% reduction in this endpoint.^12^ Of the previously published HF data from trials comparing statins to placebo or standard care, only the Heart Protection Study (HPS) demonstrated a reduction in any major HF event, namely a borderline significant reduction of 14% in the composite outcome of HF death and non-fatal HF hospitalization on simvastatin compared with placebo [354 vs. 405 first events, RR 0.86 (95% CI 0.75–1.00)].^4^ In HPS, there were also trends towards reductions in both HF death and non-fatal hospitalizations, respectively.^4,33^ However, other trials had found no such benefit, underlining the need for a comprehensive synthesis of trial data.

Notably, we found that the benefit of statin therapy for HF outcomes appeared similar regardless of whether an antecedent MI occurred or not. For example, the number of participants requiring hospitalization for non-fatal HF without a preceding within-trial MI was significantly reduced by statins, to a similar extent as that observed for participants with an earlier within-trial MI. A second notable finding was that the proportion of participants experiencing HF events was dominated by those *without* an earlier within-trial MI. Indeed, ∼85–90% of HF events were not preceded by a documented within-trial MI. There are various potential explanations for these two observations. With respect to the issue of preceding MI, both the requirement for potential events to fulfil adjudication requirements and the historical nature of the trials may have resulted in an underestimation of the numbers of within-trial MIs. Events that did not have complete documentation and the use of insensitive biomarkers may have led to this. It is also recognized that myocardial ischaemia, as well as MI, may result in reduced left ventricular systolic function and, in patients with pre-existing systolic dysfunction (which many participants in the present trials may have had), precipitate HF. For example, in the Studies of Left Ventricular Dysfunction trials, episodes of unstable angina led to a 60% increase in risk of subsequent HF hospitalization.^34^ Even myocardial ischaemia that is not acutely symptomatic, or symptomatic at all, may lead to left ventricular diastolic and systolic dysfunction (manifest in the most extreme cases as ‘hibernating myocardium’). Statins reduce symptomatic myocardial ischaemia and may also reduce asymptomatic episodes.^35^ An alternative and more controversial explanation is that statins may not only reduce the risk of developing HF by preventing ischaemic events but also by unrecognized pleiotropic mechanisms unlinked to LDL-cholesterol reduction, such as anti-inflammatory effects.^36^

The HF benefit of statin therapy is likely to be underestimated in our analysis to some extent. Our analysis was limited to hospitalization and excluded milder episodes where hospitalization was not required. Importantly, the development of HF often leads to repeated hospital admissions^1^ but our analysis was limited to the risk of experiencing a first event and not recurrent events. The duration of follow-up (4.3 years) may also have been too short to show a greater benefit. For example, the West of Scotland Coronary Prevention Study recently published data showing that, during extended follow-up of 15 years, HF hospitalization was reduced by 43% in pravastatin recipients compared with placebo recipients^37^ even though no HF benefit was observed in the initial 5 year randomized trial. We decided *a priori* to exclude HF events within 30 days after MI for reasons described above. Data from the PROVE-IT TIMI 22 study, in which participants were randomized to intensive or moderate dose statin very soon after a coronary event, suggest that this was a reasonable approach.^12^ In that trial, the effect of intensive statin therapy on risk of hospitalization for HF was very similar regardless of whether HF events in the first 30 days of randomization were included or not [hazard ratios (HRs) 0.53 and 0.55, respectively], though the effect size appeared markedly larger than observed in the present analysis.

Our analysis was designed to investigate the effect of statin therapy on the development of HF and so could not address statins' potential effect on repeat HF hospitalization in those with existing HF. However, the effect size we observed with statins was similar to that observed in the CORONA trial which compared rosuvastatin to placebo in patients with established systolic HF [5011 participants of whom 1291 were hospitalized (2408 events) for HF, HR 0.91, 95% CI 0.82–1.02]. In CORONA, statin therapy significantly reduced both the risk of repeat HF admissions and the overall number of admissions.^38^

Strengths of this meta-analysis include the following: first, it represents the largest systematic analysis evaluating the effect of statin therapy on HF events with over 132 000 participants and over 570 000 patient-years of follow-up for the main analyses. This provided power to detect potentially modest effects. Second, unpublished data were collected allowing us to provide information on homogenous HF endpoints, as far as possible. And third, access to trial data allowed harmonization of data and assessment of relevant subgroup and sensitivity analyses. Potential weaknesses include the following: first, the analyses were conducted with summary-level data, not with individual participant data, and we combined trial-specific RRs because not all trials could provide HRs for the outcomes of interest. Second, HF outcomes were not pre-specified in many trials which may have affected the quality of the data available for *post hoc* analysis, and it was not possible to use a uniform definition for HF events due to the nature of the data; however, limited heterogeneity for all the analyses provides some confidence that any such variation did not introduce systematic bias. Third, data were missing for some of the HF endpoints and not all major statin trials could be included. Fourth, we were unable to conduct a formal statistical comparison of the HF risks of participants who did and did not experience a within-trial MI prior to developing HF, but the large degree of overlap for these respective results strongly suggests that there was no difference between them. Fifth, we did not have access to data regarding the likely aetiology of HF though, based on the characteristics of the trial participants, relatively few are likely to have developed preserved ejection HF. Sixth, we were unable to compare outcomes in men and women.

In conclusion, our meta-analysis of data from major trials demonstrated that statin therapy modestly reduced the risk of non-fatal HF hospitalization and the composite outcome of HF death and non-fatal hospitalization over 4.3 years.

## Authors’ contributions

D.P. had access to all of the data in the study and takes responsibility for the integrity of the data and the accuracy of the data analysis. Study concept and design: D.P., R.T.C., J.J.M. Acquisition of data: all authors. Analysis and interpretation of data: all authors. Drafting of the manuscript: D.P., R.T.C., J.J.M. Critical revision of the manuscript for important intellectual content: all authors. Statistical analysis: D.P. Administrative, technical, or material support: none. Study supervision: J.J.M.

## Role of the sponsor

The British Heart Foundation had no role in the design and conduct of the study; the collection, analysis, and interpretation of the data; or the preparation, review, or approval of the manuscript.

## Supplementary material

Supplementary material is available at *European Heart Journal* online.

## Funding

This project was not supported by external funding. R.T.C. is funded by a BHF project grant (PG/13/17/30050). N.R.P. is supported by the National Institute for Health Research Senior Investigator Awards, Biomedical Research Centre funding, and the British Heart Foundation Research Centre Excellence Award. K.R. is supported by a National Institute for Health Research Career Development Fellowship and funding from the Oxford Biomedical Research Centre, and the Oxford Martin School. Funding to pay the Open Access publication charges for this article was provided by the University of Glasgow.

**Conflict of interest:** I.F., C.J.P., K.R., H.M.C., D.D.W., J.C.L., P.A., T.R.P., M.J.T., M.J.K., N.R.P., P.S.S., P.M.R., J.G.M., B.R.D., H.N., K.M., R.M.M., R.M., G.T., V.G.A., A.M.G., M.B.C., and J.R.D. each reported serving as an investigator in at least one of the trials. D.P. reports serving on advisory boards for Sanofi Aventis. I.F. reports receiving a research grant from Merck. C.J.P. reports receiving a research grant funding from AstraZeneca, MSD, and Roche, plus consulting for AstraZeneca, MSD, and Roche. N.S. has received consulting fees as a member of the Advisory Boards of AstraZeneca, Bristol Meyers Squibb, Amgen, and Sanofi, and received an honorarium as a speaker for MSD. H.M.C. has been a consultant or advisory panel member for Pfizer, Sanofi Aventis, Regeneron, Novartis, and Eli Lilly, has received non-binding research support from Roche, Pfizer, Eli Lilly, Boehringer Ingelheim, and AstraZeneca, has participated in a lecture/speaker's bureau and received honoraria from Pfizer, and is a shareholder in Roche. D.D.W. reports receiving remuneration for participating in clinical trial committees for Aastrom, Aegerion, Cerenis, CSL Ltd, Merck Schering-Plough, Pfizer, Shire, and Sanofi-Aventis; consulting for Anthera, Genentech, Pfizer, Roche, and Servier; and receiving speaker's honoraria from Bristol Myers Squibb, Pfizer, and Zydus Medica. J.C.L. reports consulting for Pfizer and serving on advisory groups for Amgen. P.A. reports receipt of research grant support and lecture fees from Pfizer, Sanofi, Bristol-Myers-Squibb, Merck, AstraZeneca, and Boehringer-Ingelheim, consultancy fees from Pfizer, BMS, Merck, Boehringer-Ingelheim, AstraZeneca, Bayer, Daiichi-Sankyo, Lundbeck, Edwards, Boston Scientific, Kowa, and lecture fees from Bayer, Boston Scientific, St Jude Medical. T.R.P. reports receiving speakers' honoraria from Merck (MSD), Pfizer, AstraZeneca, Roche, and Amgen; consulting for Merck (MSD) and Amgen; and receiving research grants from Merck (MSD) and Amgen. M.J.T. reports serving on advisory boards for Amgen and Aegerion; receiving a steering committee honorarium from Pfizer; receiving research grant funding and travel grants from Amgen and Pfizer. M.J.K. is an employee of a company that has received research grants for the evaluation of lipid treatments from Amgen, Eli Lilly, Pfizer, Regeneron, Roche, and Sanofi-Aventis. N.R.P. reports receiving speaker's honoraria from Pfizer and Kowa. P.S.S has served as a consultant to, received travel expenses from, and received payment for speaking at meetings for and received research funding from Pfizer. P.M.R. has received investigator initiated research grants from AstraZeneca, Novartis, Pfizer, and Amgen; serving as a consultant to Merck, ISIS, and Boston Heart; and is listed as a coinventor on patents held by the Brigham and Women's Hospital that relate to the use of inflammatory biomarkers in cardiovascular disease and diabetes that have been licensed to AstraZeneca and Siemens. S.D.S. reports receiving consulting fees and grant support from Novartis and consulting fees from Bayer. K.M. reports receiving lecture fees from Daiichi Sankyo, Merk-Banyu, Pfizer, Kowa, Astellas, Astra Zeneca, Novartis, Mitsubishi, and Tanabe. A.M.G. is on the Board of Directors of Aegerion Pharmaceuticals, Arisaph Pharmaceuticals, and Esperion Therapeutics, has been on advisory boards for Vatera Capital and Dupont, and has consulted for AstraZeneca, Janssen, Kowa, Merck, Pfizer, and Roche. M.B.C. is a consultant to, received honoraria from, and had travel/accommodations expenses covered or reimbursed by Astra Zeneca and Daiichi Sankyo. K.K.R. reports receiving honoraria for lectures or advisory boards for Pfizer, AstraZeneca, Roche, Sanofi, Regeneron, Amgen, Kowa, Aegerion, Abbott, Takeda, Novo Nordisk, and Boehringer Ingelheim, plus research grants from Pfizer, Bristol Myers Squibb, Sanofi Solvay, and Regeneron/Sanofi.
